# An In Vitro Evaluation of the Potential Neuroprotective Effects of Intranasal Lipid Nanoparticles Containing Astaxanthin Obtained from Different Sources: Comparative Studies

**DOI:** 10.3390/pharmaceutics15041035

**Published:** 2023-03-23

**Authors:** Joana Torres, José Miguel Pereira, Rita Marques-Oliveira, Inês Costa, Eva Gil-Martins, Renata Silva, Fernando Remião, Andreia Filipa Peixoto, José Manuel Sousa Lobo, Ana Catarina Silva

**Affiliations:** 1 UCIBIO, REQUIMTE, Laboratory of Pharmaceutical Technology/Centre of Research in Pharmaceutical Sciences, Faculty of Pharmacy, University of Porto, 4099-002 Porto, Portugal; 2 Associate Laboratory i4HB, Institute for Health and Bioeconomy, Faculty of Pharmacy, University of Porto, 4099-002 Porto, Portugal; 3 UCIBIO, REQUIMTE, Laboratory of Toxicology, Department of Biological Sciences, Faculty of Pharmacy, University of Porto, 4099-002 Porto, Portugal; 4 LAQV/REQUIMTE, Department of Chemistry and Biochemistry, Faculty of Sciences, University of Porto, 4099-002 Porto, Portugal; 5 FP-I3ID (Instituto de Investigação, Inovação e Desenvolvimento), FP-BHS (Biomedical and Health Sciences Research Unit), Faculty of Health Sciences, University Fernando Pessoa, 4099-002 Porto, Portugal

**Keywords:** astaxanthin, lipid nanoparticles, neurodegenerative diseases, nose-to-brain

## Abstract

The intranasal route has been suggested as a promising alternative to improve the direct transport of molecules to the brain, avoiding the need to cross the blood–brain barrier (BBB). In this area, the use of lipid nanoparticles, namely solid lipid nanoparticles (SLN) and nanostructured lipid carriers (NLC), has been highlighted as a promising strategy to improve the treatment of neurodegenerative diseases. In this work, formulations containing SLN and NLC that were loaded with astaxanthin that was obtained from different sources (astaxanthin extract (AE) from the algae *Haematococcus pluvialis* and pure astaxanthin (PA) from the fungi *Blakeslea trispora*) were prepared for nose-to-brain administration, and comparative in vitro experiments were performed to evaluate the biocompatibility of the formulations with nasal (RPMI 2650) and neuronal (SH-SY5Y) cells. Afterwards, the antioxidant activity of the formulations was evaluated for its potential neuroprotective effects, using different chemical aggressors. Finally, the cellular uptake of the astaxanthin was evaluated for the formulations that showed the greatest neuroprotection of the neuronal cells against chemical-induced damage. On the production day, all the formulations showed a particle size, a high encapsulation efficiency (EE), the presence of nanoparticles with a typical spherical shape, and a polydispersity index (PDI) and zeta potential (ZP) that are suitable for nose-to-brain administration. After three months of storage at room temperature, no significant changes were observed in the characterization parameters, predicting a good long-term stability. Furthermore, these formulations were shown to be safe with concentrations of up to 100 µg/mL in differentiated SH-SY5Y and RPMI 2650 cells. Regarding neuroprotection studies, the PA-loaded SLN and NLC formulations showed an ability to counteract some mechanisms of neurodegeneration, including oxidative stress. Moreover, when compared with the PA-loaded SLN, the PA-loaded NLC showed greater neuroprotective effects against the cytotoxicity induced by aggressors. In contrast, the AE-loaded SLN and NLC formulations showed no significant neuroprotective effects. Although further studies are needed to confirm these neuroprotective effects, the results of this study suggest that the intranasal administration of PA-loaded NLC may be a promising alternative to improve the treatment of neurodegenerative diseases.

## 1. Introduction

With the aging of the world population, neurodegenerative diseases (e.g., Alzheimer’s Disease, Parkinson’s Disease, and amyotrophic lateral sclerosis) have become a growing public health problem, as it is estimated that these diseases currently affect around 50 million people worldwide and that, by 2050, this number could rise to 115 million [1]. Although scientists have been making continuous efforts to find a therapeutically effective solution for the treatment of these diseases, no effective disease-modifying drug has yet been found and this remains an unmet medical problem [2,3]. Herein, the blood–brain barrier (BBB) remains the main obstacle to the effective management of neurodegenerative diseases, as it constitutes a barrier to the passage of most drugs that target the central nervous system (CNS) [4].

Neurons play a critical role in the normal functioning of the brain, as they are essential for the communication of nerve impulses and are distributed throughout the human body [5,6]. Thus, the progressive loss of neurons and/or their functions is the main pathophysiological hallmark underlying the progression of neurodegenerative diseases [7]. Among the common mechanisms of neurodegeneration is oxidative stress, which is a consequence of the imbalance between reactive oxygen species (ROS) production and the body’s antioxidant defences. ROS are extremely reactive free radicals that can interact with cell membranes, triggering cell degeneration, and, consequently, cell death. It is noteworthy that oxidative stress interferes with several cellular functions and leads to many pathological conditions, including aging, asthma, arthritis, autoimmune diseases, cardiovascular dysfunction, carcinogenesis, cataract, diabetes, and neurodegenerative diseases. To counteract this process, the body produces several molecules with antioxidant activity that is either involved in enzymatic antioxidant defences (e.g., superoxide dismutase (SOD), glutathione peroxidase (GPx), and catalase) or involved in non-enzymatic defences (e.g., glutathione, vitamin C, and vitamin E). However, sometimes, these antioxidant defences are not enough to avoid oxidative stress, and, consequently, to avoid neurodegeneration. It is also noteworthy that several studies have reported the benefits of antioxidant supplementation to delay the process of aging, apoptosis, and the progression of neurodegenerative diseases [8,9].

Thereby, the search for improved treatments for neurodegenerative diseases has involved the use of antioxidants, and many compounds have been studied for this purpose [10]. In addition, the biotechnology industry has taken into account the demand for antioxidant compounds that are obtained from natural sources to replace artificial antioxidants, such as butylhydroxytoluene (BHT) and butylhydroxyanisole (BHA), whose safety profiles are increasingly controversial due to their associated risks of carcinogenesis and liver toxicity. In recent years, natural antioxidant molecules that have been extracted from bioresources have been replacing synthetic antioxidants [11,12,13].

Marine organisms and their bio-waste represent a vast source of bioactive compounds with neuroprotective antioxidant effects, whose use has been suggested as promising in the management of neurodegenerative diseases [14,15]. Among these compounds, carotenoids are the most promising and most studied, due to their potent antioxidant activity that provides the neutralization of oxidative stress [16,17,18,19]. In particular, recent studies have reported that astaxanthin, a carotenoid found in the marine environment, has a preventive effect on neurodegenerative diseases that are induced by oxidative stress [10,20,21].

Astaxanthin, or 3,3′-dihydroxy-β,β′-carotene-4,4′-dione, is a red carotenoid with potent antioxidant activity, which can be 10 times greater than that of other carotenoids and 500 times greater than that of α-tocopherol [22]. The most common sources of astaxanthin are the microalgae *Haematococcus pluvialis*, the yeast *Phaffia rhodozyma* (*Xanthophyllomyces dendrorhous*), the bacteria *Agrobacterium aurantiacum* and *Bacillus circulans*, and some plants, such as *Adonis* sp. This compound can also be obtained from marine bio-waste, including crustacean shells, supporting the blue bioeconomy growth [23,24,25].

Several investigations have suggested that astaxanthin is able to counteract distinct pathophysiological mechanisms in patients with neurodegenerative diseases, revealing its promising potential in preventing the development/progression of these diseases [16,26,27,28]. Notably, this compound has been shown to have a cytoprotective effect, preventing the neuronal death that is induced by the oxidative stress resulting from high levels of ROS [16,21,28,29]. Astaxanthin exhibits cytoprotection due to its powerful antioxidant activity and its ability to cross lipid bilayers, providing internal and external cellular protection against oxidative stress, unlike most antioxidants, which act only inside or outside the cells [10,27]. It has also been reported that astaxanthin suppresses the mitochondrial dysfunction that is induced by oxidative stress, which further originates the mitochondrial ROS production [20].

As mentioned, the development of innovative strategies for the management of neurological diseases is challenging. In this area, different approaches have been investigated and it seems that the use of lipid-based nanosystems, such as solid lipid nanoparticles (SLN) and nanostructured lipid carriers (NLC), is among the most promising, as they are well-known nanosystems whose use has been proposed for several administration routes. Among the most important properties of these systems is their effectiveness in protecting lipophilic molecules, providing prolonged release, and promoting drug absorption [30,31,32,33]. To improve the management of neurodegenerative diseases, the direct nose-to-brain delivery of lipid-based nanosystems has gained the interest of several researchers, as it avoids the need of crossing the BBB in order for the molecules to reach the brain [34]. Nasal/intranasal administration offers advantages over parenteral routes, including non-invasiveness, the ease of application, and an absence of pain. Furthermore, through the nose, the molecules can be absorbed directly into the bloodstream, escaping the gastrointestinal inactivation and hepatic metabolism that can occur with oral administration [34,35,36]. Nonetheless, other lipid-based nanosystems, such as liposomes [37] and nanoemulsions [38], have demonstrated an effectiveness in improving the oral delivery of lipophilic and hydrophilic natural antioxidants.

The aim of this work was to encapsulate the astaxanthin that was obtained from different sources (astaxanthin extract (AE) from the algae *Haematococcus pluvialis* and pure astaxanthin (PA) from the fungi *Blakeslea trispora*) in SLN and NLC. These formulations were planned to be administered intranasally, aiming the direct transport of the compound from the nose to the brain. The experiments consisted of comparative studies on the biocompatibility of astaxanthin-loaded and empty SLN and NLC formulations, assessed in nasal (RPMI 2650) and neuronal (SH-SY5Y) cells. Afterwards, the antioxidant activity of these formulations was evaluated and compared for its potential neuroprotective effects, after exposing cells to different chemical aggressors, such as iron (III) (in the form of an iron (III)–nitrilotriacetic acid (NTA) complex (FeNTA) to simulate iron overload), *t*-BHP (to induce oxidative stress), and MPP^+^ (the active metabolite of MPTP, used in vitro to mimic Parkinson’s Disease). Finally, the cellular uptake of the astaxanthin was evaluated for the formulations that showed the greatest neuroprotection of the neuronal cells against chemical-induced damage.

## 2. Materials

The astaxanthin extract (AE) containing ≥10% of astaxanthin was obtained from the *Haematococcus pluvialis* algal biomass, ≤0.8% D-α-tocopherol, 0–30% high oleic sunflower oil was kindly donated from Algalif (Bogatrod, Iceland), and the pure astaxanthin (PA) (containing ≥ 97% of astaxanthin) that was produced from the fungi *Blakeslea trispora* was purchased from Sigma (Lisbon, Portugal). Vitamin E, benzalkonium chloride, Tween^®^ 80 (polysorbate 80), and glycerine were acquired from Acofarma (Madrid, Spain). Sodium deoxycholate was obtained from Sigma (Lisbon, Portugal). Precirol^®^5ATO (glyceryl distearate) was kindly donated from Gatefossé (Neuilly sur-Seine, France). Dulbecco’s modified eagle medium (DMEM)–high glucose, trypsin–ethylenediamine tetraacetic acid (EDTA) solution (0.25% trypsin/1 mM EDTA), and Triton^TM^ X100 were obtained from Thermo Fisher Scientific (Waltham, MA, USA). Eagle’s minimum essential medium (EMEM) with L-glutamine was acquired from the American Type Culture Collection (ATCC) (Rockville, MD, USA).

The human neuroblastoma SH-SY5Y and the human nasal septum carcinoma RPMI 2650 cell lines were obtained from ATCC (Rockville, MD, USA). Neutral red (NR) solution, 1-methyl-4-phenylpyridinium (MPP^+^), sodium deoxycholate, 3-(4,5-dimethylthiazol-2-yl)-2,5-diphenyl tetrazolium bromide (MTT), iron (III) chloride (FeCl_3_), nitrilotriacetic acid disodium salt (Na_2_NTA), nitrilotriacetic acid trisodium salt (Na_3_NTA), tert-Butyl hydroperoxide (*t*-BHP), retinoic acid (RA), phorbol 12-myristate 13-acetate (TPA), and trans-β-apo-8′-carotenal were obtained from Sigma-Aldrich (Schnelldorf, Germany). Hanks’ balanced salt solution (HBSS), with or without calcium and magnesium [HBSS (+/+) or HBSS (−/−), respectively, heat-inactivated fetal bovine serum (FBS), and non-essential amino acids were obtained from PAN-Biotech (Aidenbach, Germany).

Dimethyl sulfoxide (DMSO) was obtained from Merck (Darmstadt, Germany). Antibiotic mixture (10,000 U/mL penicillin, 10,000 μg/mL streptomycin) was obtained from Biochrom (Berlin, Germany). Glacial acetic acid was purchased from Fischer Scientific (Lisbon, Portugal). Methanol and ethanol were acquired from Honeywell (Charlotte, NC, USA). All the sterile plastic material that was used was acquired from Corning Costar (Glendale, AZ, USA). The purified water that was used in all the experiments was obtained from a Direct-Q^®^ Ultrapure Water Systems, Merck Millipore (Darmstadt, Germany). All the reagents that were used were of analytical grade or of the highest grade available.

## 3. Methods

### 3.1. Development and Characterization of SLN and NLC Formulations Containing Astaxanthin

#### 3.1.1. Evaluation of Compatibility between Astaxanthin and Lipids

An evaluation of the solubility of the AE and PA in the lipids that were used to prepare the SLN and NLC formulations is fundamental, as it represents their capacity to encapsulate a high amount of astaxanthin and identify the ideal ratio between the compound and lipids that leads to the formation of a solid matrix [39].

The lipids that were used to prepare the SLN and NLC formulations were selected from the previous works that were developed by our group [40]. Precirol^®^ ATO5 was used as the solid lipid, due to its melting point above room temperature (~56 °C) and ability to form the solid lipid matrix of both SLN and NLC. Vitamin E was selected as the liquid lipid, due to its antioxidant activity that may potentially contribute to the delayed neuronal damage caused by oxidative stress, providing NLC formulations with a greater neuroprotective effect. In addition, its presence reduces the risk of lipid oxidation when preparing formulations and facilitates the solubilisation of lipophilic molecules, which is an advantage in the case of astaxanthin, as it is a very lipophilic molecule [41].

The solubility of the astaxanthin in Precirol^®^ ATO 5 was evaluated for concentrations ranging from 0.5 to 1% (*w*/*w*) of the compound in the lipid. For the experiments, different amounts of AE or PA were added to the solid lipid. Then, the mixtures were heated at 80 ± 1 °C and stirred (200 rpm) for 1 h. The existence of the solubility between the astaxanthin and the lipid was evaluated macroscopically, observing the presence or absence of lipid crystals, at time intervals of 15 min. Afterwards, to study the solubility of the astaxanthin in vitamin E, different amounts of AE or PA (0.5 to 1%) were added to the liquid lipid and the mixture was vigorously stirred, observing the occurrence of miscibility [40].

#### 3.1.2. Preparation of SLN and NLC Formulations

The ultrasound technique that was previously developed by Silva et al. [40,42] was used to produce the SLN and NLC formulations. Briefly, the lipid phase was heated at 5–10 °C above the melting point of the solid lipid. Simultaneously, the aqueous phase was heated to the same temperature on a heating plate. After the melting and checking that both phases were at the same temperature, the aqueous phase was added to the lipid phase. This mixture was immediately emulsified with an Ultra-turrax^®^ T25 (Janke and Kunkel GmbH, Staufen, Germany) at 13.400 rpm for 16 min. The oil-in-water (O/W) emulsion that was obtained was subjected to sonication energy for 20 min, at an amplitude of 50%, using an ultrasonic probe (VCX 130 ultrasonic processor, Sonics, Newtown, CT, USA). The O/W nanoemulsion that was obtained was transferred to glass vials, which were immediately placed on an ice bath to form the SLN or the NLC. All the materials that were used for the preparation and storage of the PA- and AE-loaded SLN and NLC formulations were protected from the light to avoid compound degradation [27].

Table 1 shows the composition of the developed SLN and NLC formulations, empty and loaded with AE and PA.

#### 3.1.3. Particle Size, Polydispersity Index (PDI), and Zeta Potential (ZP)

The developed NLC and SLN formulations, empty and loaded with AE and PA, were characterized for their particle size, PDI, and ZP.

The particle size was assessed by dynamic light scattering (DLS) in a Nanozetasizer (Malvern Panalytical, Worcestershire, United Kingdom). The PDI and ZP were also evaluated in the Nanozetasizer. The samples were first diluted in ultrapure water (1:100) and the measurements were performed using an absorption index of 0.001, a particle refractive index of 1.6, and ultrapure water as dispersing medium.

The measurements were performed in triplicate (*n* = 3) and the results were presented as mean values ± SD. The system software, using the Helmholtz–Smoluchowski equation, calculated the ZP values [42,43,44].

The long-term stability of the developed SLN and NLC formulations was assessed after 0, 30, and 180 days of storage at 4.00 ± 0.5 °C and 20.0 ± 0.5 °C. The formulations were characterized by their particle size, PDI, and ZP. A visual observation of colour changes, aggregation, and flocculation, which were indicative of the occurrence of instability phenomena, was also recorded [40].

#### 3.1.4. Evaluation of the Encapsulation Efficiency (EE)

The EE of the astaxanthin in the NLC and SLN formulations was assessed indirectly, through measuring the amount of non-encapsulated compound. For this purpose, the AE- or PA-loaded SLN and NLC were diluted in ethanol, passed into an Amicon^®^ Ultra-4 (Millipore Corporation, Darmstadt, Germany) and centrifuged for 1 h at 3500 rpm. Afterwards, the supernatant was collected, filtered using a 0.22 μm cellulose acetate filter (FilterBio^®^ PTFE Syringe Filter, Lisbon, Portugal), diluted 20× in methanol containing 1 μg/mL of internal standard (*trans*-*β*-Apo-8′-carotenal), and finally injected into a HPLC-UV apparatus. All the measurements were carried out in triplicate (mean ± SD), at 20.0 ± 3.0 °C.

The quantification of the astaxanthin was performed in a HPLC Chromaster (VWR International, Lisbon, Portugal), equipped with a Hitachi Chromaster 5160 pump, a Hitachi Chromaster 5260 autosampler, and a Hitachi Chromaster 5310 column oven, and coupled to a Hitachi Chromaster 5410 UV detector. A C18 ACE column with a 5 μm diameter and 250 × 4.6 mm was used. The analysis was performed in isocratic elution mode, using 97:3 (*v*/*v*) of methanol/water as the mobile phase and a flow rate of 1 mL/minute. The mobile phase was previously filtered using a 0.45 μm membrane (Millipore Corporation, Darmstadt, Germany). The sample injection volume was 20 μL and the UV detection was performed at 474 nm. The Clarity^®^ Software 7.0 was used for the data analysis.

The *EE* was calculated according to the following equation [45]:

$$EE\;(\%)=\frac{Total\;amount\;of\;astaxanthin\;-\;Free\;astaxanthin}{Total\;amount\;of\;astaxanthin}\;\times \;100$$

#### 3.1.5. Cryo-Scanning Electron Microscopy (cryoSEM)

The analysis of the surface shape and morphology of the SLN and NLC was performed with cryoscanning electron microscopy (cryoSEM), using a JSM-6301F (JEOL, Tokyo, Japan). Prior to analysis, the sample was frozen in slush nitrogen, fractured, sublimated for 120 s at −90 °C, and coated with gold and palladium for 45 s at 12 mA. Finally, the sample was transferred into the SEM chamber and observed at −150 °C [39,40,44].

### 3.2. Biocompatibility Studies

The biocompatibility of the developed NLC and SLN formulations, empty and loaded with PA or AE, was assessed in differentiated SH-SY5Y cells and RPMI 2650 cells, using the NR uptake and MTT reduction assays, 24 h after exposure. The cytotoxicity of the free PA and AE, in the same concentration range that was found in the PA- or AE-loaded NLC and SLN, was also assessed. Triton™ X-100 (0.1%) was used as a positive control.

#### 3.2.1. Cell Culture

##### SH-SY5Y Cells

SH-SY5Y is a neuroblastoma cell line that is cloned from SK-N-SH cells and has been extensively used as a neuronal model, as these cells hold numerous biochemical and functional properties of neurons, including the ability to transport dopamine and noradrenaline, the expression of enzymes that are involved in the synthesis and metabolism of dopamine and acetylcholine, and the expression of dopamine and acetylcholine receptors. Although SH-SY5Y cells can be used in their undifferentiated form, different protocols are applied to differentiate these cells, obtaining, for example, SH-SY5Y cells with a dopaminergic phenotype. Indeed, the use of undifferentiated SH-SY5Y cells as an in vitro model can present some limitations, such as a low expression of the enzymes involved in the synthesis of dopamine and a continuous mitosis process that can affect the response to drugs and toxins. It was demonstrated that differentiating these SH-SY5Y cells with retinoic acid, followed by treatment with phorbol 12-myristate 13-acetate, significantly improved these aspects [46,47].

The SH-SY5Y cells were routinely cultured in 25 cm^2^ flasks using DMEM–high glucose, and were supplemented with 10% heat-inactivated FBS, 1% MEM non-essential amino acids, and 1% antibiotic mixture (100 U/mL of penicillin and 100 μg/mL of streptomycin). The cells were kept in a 5% CO_2_ 95% air atmosphere at 37 °C, and the medium was changed every 2–3 days. The cultures were passaged weekly by trypsinization, using a 0.25% trypsin- 1mM EDTA solution. For each experiment, the cells were seeded in 96-well plates at a density of 25.000 cells/cm^2^. For the cell differentiation into a dopaminergic phenotype, they were seeded in a complete DMEM medium containing retinoic acid (final concentration of 10 μM). After 3 days, phorbol 12-myristate 13-acetate (final concentration of 80 nM) was added for more 3 days, and the cells were used 6 days after seeding. Six days after seeding, the cells were exposed to the NLC and SLN formulations (empty or loaded with PA or AE, 0–500 μg/mL), as well as exposed to the free AE or PA, in the same concentration range that was found in the AE- and PA-loaded formulations, for 24 h. For the in vitro studies, a 25 g/L PA or AE stock solution was prepared in DMSO and stored at −20 °C. On the day of the experiment, the NLC and SLN formulations (empty, PA-, or AE-loaded) and the free AE and PA were diluted in a fresh cell culture medium to obtain the desired final concentrations. The cells that were used in all the experiments were taken between the 20th and 25th passages.

##### RPMI 2650 Cells

RPMI 2650 cells are isolated from a squamous cell carcinoma of the human nasal septum. In experiments with nasal formulations, the RPMI 2650 is the primary human nasal cell line that is used, as the use of this in vitro model represents a non-invasive method for predicting nasal drug delivery and shows a reproducibility of results. RPMI 2650 cells can reach confluence in a short time period, as they have a consistent growth rate. Thereby, the use of these cells is preferred over tests on excised nasal tissue, as they are less expensive and easier to maintain. However, although RPMI 2650 cells create tight junctions with transepithelial electrical resistance, mimicking the nasal mucosa, and can secrete a consistent coating of mucus onto the cell surface, they are unable to perform the mucociliary clearance mechanism, as they do not contain cilia [48,49,50].

The RPMI 2650 cells were routinely cultured in 75 cm^2^ flasks using EMEM with L-glutamine, and ertr supplemented with 10% heat-inactivated FBS and 1% antibiotic mixture (100 U/mL penicillin and 100 μg/mL streptomycin). The cells were kept in a 5% CO_2_ 95% air atmosphere at 37 °C, and the medium was changed every 1–2 days. The cultures were passaged weekly by trypsinization using a 0.25% trypsin-1 mM EDTA solution. For each experiment, the cells were seeded in 96-well plates at a density of 160.000 cells/cm^2^ In all the experiments, the cells were used 24 h after seeding. The cells that were used in all the experiments were taken between the 5th and 10th passages.

For the experiments, 24 h after seeding, the cells were exposed to the NLC and SLN formulations (empty or loaded with PA or AE, 0–500 μg/mL), as well as exposed to the free AE or PA, in the same concentration range that was found in the astaxanthin-loaded formulations, for 24 h. On the day of the experiment, the NLC and SLN formulations (empty, PA-, and AE-loaded) and the free AE and PA were diluted in a fresh cell culture medium to obtain the desired final concentrations.

#### 3.2.2. MTT Reduction

The MTT reduction assay is based on the conversion of the yellow water-soluble tetrazolium salt, MTT, into purple water-insoluble formazan crystals, which is achieved by the mitochondrial dehydrogenases of viable cells. Thus, it represents a simple colorimetric assay for assessing cell mitochondrial activity, being the amount of formed formazan crystals that are directly proportional to the amount of metabolic viable cells in a culture, thus being an effective and easy method of assessing cellular cytotoxicity [51,52].

The MTT reduction assay was performed, as previously described [53]. Briefly, the cell culture medium was removed at the chosen time point (24 h), and the cells were further incubated for 1.5 h in a humidified 5% CO_2_ 95% air atmosphere, at 37 °C, with 150 μL of 0.5 mg/mL MTT that was prepared in a fresh cell culture medium. Afterwards, the cell culture medium was removed, the formed formazan crystals were solubilized with 150 μL of DMSO, and the absorbance was then measured at 550 nm in a multiwell plate reader (PowerWaveX BioTek Instruments, Winooski, VT, USA). The cytotoxicity of the developed NLC and SLN formulations (empty and loaded with PA or AE), as well as the cytotoxicity of the free PA or AE, was evaluated as the percentage of the MTT reduction when compared to the control cells (0 μg/mL). At least four independent experiments (*n* = 4) were performed in triplicate.

#### 3.2.3. Neutral Red Uptake

The neutral red (NR) uptake is a colorimetric assay that is based on the ability of viable cells to incorporate and bind the cationic NR dye into their lysosomes, thus allowing for a rapid assessment of the cell viability [54]. Indeed, after penetrating the cell membrane, through non-ionic passive diffusion, NR establishes electrostatic interactions with phosphate groups and/or anionic sites. The characteristic lysosome proton gradient allows the NR to become charged and kept in the lysosomal matrix. However, the ability of the cells to maintain a pH gradient directly affects the NR uptake, as there is no dye retention in the case of a pH gradient reduction or cell death. Therefore, the amount of the NR that is incorporated within the lysosomes is directly proportional to the number of viable cells in a culture. Moreover, the NR uptake is also influenced by damages in the lysosomal membrane or by differences in the cell surface, thus allowing for a distinction between damaged, dead, and viable cells [55].

The NR uptake assay was performed, as previously described [40]. Briefly, at the chosen time point (24 h), the cell culture medium was removed and the cells were further incubated for 1.5 h at 37 °C, and in a humidified 5% CO_2_ 95% air atmosphere, with 50 μg/mL of the NR prepared in a fresh cell culture medium. Afterwards, the cell culture medium was removed and the NR that was incorporated in the lysosomes of the viable cells was then extracted with 150 μL of absolute ethanol/distilled water (1:1) with 5% glacial acetic acid. The absorbance was then measured at 540 nm in a multiwell plate reader (PowerWaveX BioTek Instruments, Winooski, VT, USA). The cytotoxicity of the developed NLC and SLN formulations (empty and loaded with PA or AE), as well as the cytotoxicity of the free PA or AE, was evaluated as the percentage of the NR uptake when compared to the control cells (0 μg/mL). At least four independent experiments (*n* = 4) were performed in triplicate.

### 3.3. Evaluation of the Neuroprotective Effects

The potential neuroprotective effects of the developed PA- or AE-loaded NLC and SLN formulations, the empty NLC and SLN, and the free PA and AE, were assessed in the differentiated SH-SY5Y cells. For this purpose, different chemical aggressors, such as *t*-BHP (to induce oxidative stress), MPP^+^ (the active metabolite of MPTP, used in vitro to mimic Parkinson’s Disease), and iron (III) (in the form of an iron (III)–nitrilotriacetic acid (NTA) complex (FeNTA) to simulate iron overload) were tested.

#### 3.3.1. Protective Effects against *t*-BHP-Induced Cytotoxicity

The imbalance between the ROS formation and the antioxidant defence, in favour of increased ROS levels, is the definition of oxidative stress. Consequently, the vital constituents of cells, including lipids, proteins, and nucleic acids, are oxidized by the ROS, which induce cell damage and apoptosis or necrosis. As oxidative stress is one of the main causes underlying several neurodegenerative diseases, *t*-BHP is a ROS inducer that can be used in vitro to identify the drugs with potential antioxidant neuroprotective effects [56].

In this assay, the SH-SY5Y cells were seeded at a density of 25.000 cells/cm^2^ into 96-well plates, and further submitted to the differentiation protocol that is mentioned in the SH-SY5Y Cells Section above. Six days after seeding, the cells were exposed to the PA-loaded or AE-loaded NLC and SLN formulations (50 and 100 µg/mL), and to the respective concentrations of the free PA or AE, prepared in a fresh cell culture medium. The empty NLC and SLN formulations (50 and 100 µg/mL) were also tested. After 30 min of pre-incubation, *t*-BHP (final concentration of 75 µM) was added and the cells were further incubated at 37 °C, in a humidified 5% CO_2_ 95% air atmosphere for 24 h. After this incubation, the *t*-BHP cytotoxicity was evaluated by the NR uptake assay, as described before (Section 3.2.3). For each experiment, a fresh stock solution of *t*-BHP was prepared (protected from light) in a fresh cell culture medium. At least four independent experiments (*n* = 4) were performed in triplicate.

#### 3.3.2. Protective Effects against MPP^+^-Induced Cytotoxicity

One of the pathological hallmarks of Parkinson’s Disease is the loss of dopaminergic neurons in the substantia nigra pars compacta. To replicate this disease in vitro, the MPP^+^ neurotoxin, the active metabolite of MPTP, is extensively used, as it is capable of severely damaging neuronal cells and inducing cell death, thus closely mimicking Parkinson’s Disease [57,58,59]. MPP^+^ is responsible for the inhibition of complex I of the mitochondrial electron transport chain, leading to ROS formation and ATP depletion, which changes the mitochondrial membrane potential and leads to apoptosis and the death of dopaminergic neurons [60,61]. Therefore, the cytotoxicity that is induced by MPP^+^ has been frequently used to find the agents with potential neuroprotective effects [59].

In this assay, the SH-SY5Y cells were seeded at a density of 25,000 cells/cm^2^ into 96-well plates and submitted to the differentiation protocol that is mentioned in the SH-SY5Y Cells Section above. Six days after seeding, the cells were exposed to the PA-loaded or AE-loaded NLC and SLN formulations (50 and 100 µg/mL), and to the respective concentrations of the free PA or AE, prepared in a fresh cell culture medium. The empty NLC and SLN formulations (50 and 100 µg/mL) were also tested. After 30 min of pre-incubation, MPP^+^ (final concentration of 1000 µM) was added and the cells were further incubated at 37 °C, in a humidified 5% CO_2_ 95% air atmosphere for 24 h. After this incubation, the MPP^+^ cytotoxicity was evaluated by the NR uptake assay, as described before (Section 3.2.3). In each experiment, a fresh stock solution of MPP^+^ (10 mM) was prepared (protected from light) in HBSS (+/+), diluted in a fresh cell culture medium, and immediately used. At least four independent experiments (*n* = 4) were performed in triplicate.

#### 3.3.3. Protective Effects against Iron (III)-Induced Cytotoxicity

The protective effects of the PA-loaded NLC and SLN formulations, and of the free PA, were further evaluated against iron overload, a pathophysiological hallmark that is observed in several neurodegenerative diseases. To assess the neuroprotective effects against the cytotoxicity that is induced by ferric iron, FeNTA was chosen as an iron aggressor. Indeed, for investigational purposes, FeNTA stops the hydrolysis of iron (III) at physiological pH and has been extensively used as an iron source for many biological studies, as the NTA reasonable affinity for iron makes FeNTA an admirable model for imitating the small ligands that are involved in metal-chaperone proteins [62]. The FeNTA concentration that was tested (1000 µM) was selected based on previous works [59,63,64].

Firstly, the NTA (250 mM, pH 7.4) solution was set by mixing a nitrilotriacetic acid disodium salt (Na_2_NTA) solution (100 mM) and a nitrilotriacetic acid trisodium salt (Na_3_NTA) solution (100 mM) until the pH = 7. Iron (III) chloride was then combined with the NTA to obtain the FeNTA solution. For each experiment, a fresh FeNTA solution (100 mM) was prepared (protected from light) and incubated for 15 min, in order to assure the complete development of the ferric oxidation form before adding the solution to the cells [59,65]. The stock FeNTA solution was further diluted in a fresh cell culture medium to obtain the desired final working concentration.

For this assay, the SH-SY5Y cells were seeded at a density of 25,000 cells/cm^2^ into 96-well plates and submitted to the differentiation protocol that is described in the SH-SY5Y Cells Section above. Six days after seeding, the cells were exposed to the PA-loaded NLC and SLN formulations (50 and 100 µg/mL), and to the respective concentrations of the free PA (0.378 and 0.756 µg/mL for 50 and 100 µg/mL PA-loaded SLN, respectively, and 0.367 and 0.734 µg/mL for 50 and 100 µg/mL PA-loaded NLC, respectively), and were prepared in a fresh cell culture medium. The empty NLC and SLN formulations (50 and 100 µg/mL) were also tested. After 30 min of pre-incubation, the FeNTA (final concentration of 1000 µM) was added, and the cells were further incubated at 37 °C, in a humidified 5% CO_2_ 95% air atmosphere for 24 h. After this incubation, the FeNTA cytotoxicity was assessed by the NR uptake assay, as described before (Section 3.2.3). At least four independent experiments (*n* = 4) were performed in triplicate.

### 3.4. Astaxanthin Cellular Uptake

According to the results of the neuroprotective effects (see Section 5.3), the astaxanthin uptake was assessed in the differentiated SH-SY5Y cells. For the experiments, the SH-SY5Y cells were seeded in 6-well plates at a density of 25,000 cells/cm^2^, and submitted to the RA and TPA differentiation protocol that was mentioned before. Six days after seeding, the SH-SY5Y cells were exposed to the PA-loaded SLN and NLC formulations (100 μg/mL), as well as to the free PA at the respective astaxanthin concentrations (0.756 μg/mL for 100 μg/mL PA-loaded SLN and 0.734 μg/mL for 100 μg/mL PA-loaded NLC). The cell culture medium was removed 24 h after exposition and 250 μL of methanol, containing 1 μg/mL of internal standard (*trans*-*β*-Apo-8′-carotenal), was added to each well for 30 min, to lysis the cells and extract the intracellular astaxanthin content. After this incubation, the plates were centrifuged at 250× *g* at 4 °C, for 5 min, and the methanol with the internal standard was collected and injected (20 µL) in an HPLC-UV to quantify the amount of astaxanthin in the samples. The chromatographic conditions that were used were the ones that are described in Section 3.1.4. Finally, 250 μL of NaOH was added to each well of the plate to solubilize the protein content and was left, protected from the light, at 4 °C for 24 h. Afterwards, the protein samples were stored at −20 °C until the analysis, which was made using the DC Protein Assay Kit from BioRad (Lisbon, Portugal). The intracellular astaxanthin content in the samples was normalized to the protein content and the results were expressed as the ng of astaxanthin per mg of protein. At least four independent experiments (*n* = 4) were performed in triplicate.

## 4. Statistical Analysis

The statistical calculations were executed using GraphPad Prism 8 for Windows (GraphPad Software 8, San Diego, CA, USA). The normality of the data distribution was measured using the Kolmogorov–Smirnov, D’Agostino and Pearson omnibus, and Shapiro–Wilk normality tests. In the biocompatibility studies, one-way ANOVA was used to perform the statistical comparisons, followed by the Dunnett’s multiple comparisons test. The statistical comparisons between the groups in the experiments with two variables (neuroprotection studies) were made using two-way ANOVA, followed by Šídák’s multiple comparisons test (for comparisons to the control cells (0 µM of chemical aggressor)), or by Tukey’s multiple comparison post hoc test (for comparisons between the astaxanthin-loaded formulations (PA or AE-loaded SLN and NLC), the free AE or PA, the empty SLN and NLC, and the chemical aggressor alone, at each aggressor concentration). In the evaluation of the astaxanthin cellular uptake, an unpaired *t* test was used to perform the statistical comparisons. The details of the performed statistical analyses are described in the figure legend. In all the experiments, the *p* values under 0.05 were considered to be statistically significant.

## 5. Results

### 5.1. Development and Characterization of SLN and NLC Formulations Containing Astaxanthin

#### 5.1.1. Evaluation of Compatibility between Astaxanthin and Lipids

The results of the solubility studies of the astaxanthin (AE and PA) in Precirol^®^ ATO 5 and vitamin E showed that all the tested ratios were miscible, and that no crystals that were characteristic of immiscibility were detected. Accordingly, the maximum amount of the AE and PA that were tested (1%) was selected to prepare the SLN and NLC (Table 1). Since the solubility of the active compounds in the lipids influences the ability of the SLN and NLC to encapsulate molecules, these results predict a high EE [39,66,67].

#### 5.1.2. Particle Size, Polydispersity Index (PDI), and Zeta Potential (ZP)

The results of the particle size (Z-Ave), PDI, and ZP of the developed SLN and NLC formulations are shown in Table 2.

In Table 2, it can be seen that, on the day of production, AE_SLN had an average size of 106.967 ± 2.515 nm, PA_SLN had an average size of 110.200 ± 6.482, and the empty SLN had an average size of 98.043 ± 1.200 nm. Regarding the sizes of the NLC formulations on day 0, it was observed that AE_NLC had an average size of 117.300 ± 2.163 nm, PA_NLC had an average size of 97.610 ± 0.394, and the empty NLC had an average size of 109.033 ± 0.404 nm.

In addition, from Table 2, it is also possible to conclude that the empty SLN and NLC formulations, and those with AE and PA, were monodisperse, as all the PDI values were smaller than 0.300 [68,69]. In relation to the ZP, all the formulations presented values close to |30| mV, suggesting long-time stability [70].

After 180 days of the preparation of the SLN and NLC formulations, and when stored at 20.0 ± 0.5 °C (Table 2), the empty SLN, empty NLC, AE_SLN, and AE_NLC had sizes of 98.107 ± 0.641, 116.033 ± 1.795, 104.367 ± 0.404, and 174.167 ± 1.210, respectively. In addition, when stored at 4.0 ± 0.5 °C, the particle size values were slightly larger, although this increase was not significant, as the nanoparticles maintained an adequate size for nose-to-brain delivery. However, PA_SLN and PA_NLC showed macroscopic changes of aggregation and flocculation after 180 days of storage and after 30 days of storage at 4.0 ± 0.5 °C, although these formulations were found to show stability up to 30 days of storage at room temperature (20.0 ± 0.5 °C). Accordingly, the PDI and ZP results also showed that all the empty SLN, empty NLC, AE_SLN, and AE_NLC that were stored for over 180 days at 4.0 ± 0.5 °C and 20.0 ± 0.5 °C were still monodisperse, with PDI values lower than 0.300, and stable, with ZP values close to |30| mV.

#### 5.1.3. Evaluation of the Encapsulation Efficiency (EE)

The EEs of the EA and PA were, respectively, 99.99 ± 0.00% and 98.75 ± 0.88% for the SLN, and 99.61 ± 0.04% and 98.55 ± 0.39% for the NLC.

#### 5.1.4. Cryo-Scanning Electron Microscopy (cryoSEM)

**Figure 1:**
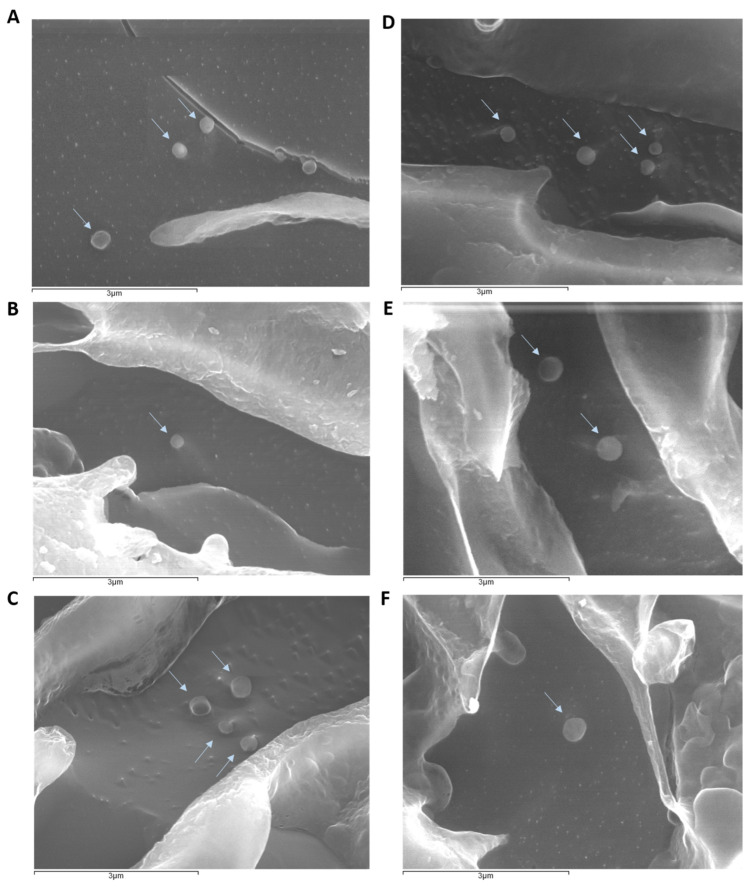
CryoSEM images of the developed SLN and NLC formulations. White arrows indicate the nanoparticles. (**A**) empty SLN (magnification 20.000×); (**B**) AE_SLN (magnification 20.000×); (**C**) PA_SLN (magnification 20.000×); (**D**) empty NLC (magnification 20.000×); (**E**) AE_NLC (magnification 20.000×); and (**F**) PA_NLC (magnification 20.000×).

Figure 1 shows that both the NLC and SLN (empty and loaded with AE or PA) had a similar appearance and the typical spherical shape and plane surface of lipid nanoparticles [26]. In addition, it is possible to confirm the existence of particles with nanometric sizes, as presented in Table 2. Similar studies with astaxanthin-loaded lipid nanoparticles have exhibited identical images to those obtained in this work [26,29].

### 5.2. Biocompatibility Studies

The cytotoxicity of the empty SLN and NLC, and the AE- and PA-loaded SLN and NLC formulations (0–500 µg/mL), as well as the cytotoxicity of the free AE and PA, was evaluated in the differentiated SH-SY5Y cells and RPMI 2650 cells, via the MTT reduction and NR uptake assays, 24 h after exposure.

#### 5.2.1. SH-SY5Y Cells

**Figure 2:**
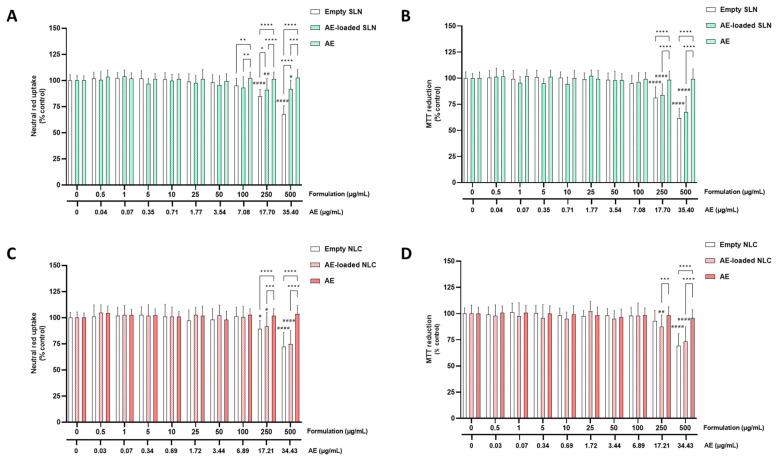
Cytotoxicity of the AE-loaded SLN and NLC, the corresponding concentrations of free AE, and empty SLN and NLC formulations, evaluated in differentiated SH-SY5Y cells by the neutral red uptake (**A**,**C**) and MTT reduction (**B**,**D**) assays, 24 h after exposure. Results are expressed as mean + SD from at least 4 independent experiences, performed in triplicate. Statistical comparisons were made using two-way ANOVA to compare means of different groups, followed by the Tukey’s multiple comparisons test (^#^ *p* < 0.05; ^##^ *p* < 0.01; and ^####^ *p* < 0.0001 for each formulation vs. 0 μg/mL; * *p* < 0.05; ** *p* < 0.01; *** *p* < 0.001; and **** *p* < 0.0001 for comparisons between formulations, at each concentration). In all cases, *p* values < 0.05 were considered significant.

**Figure 3:**
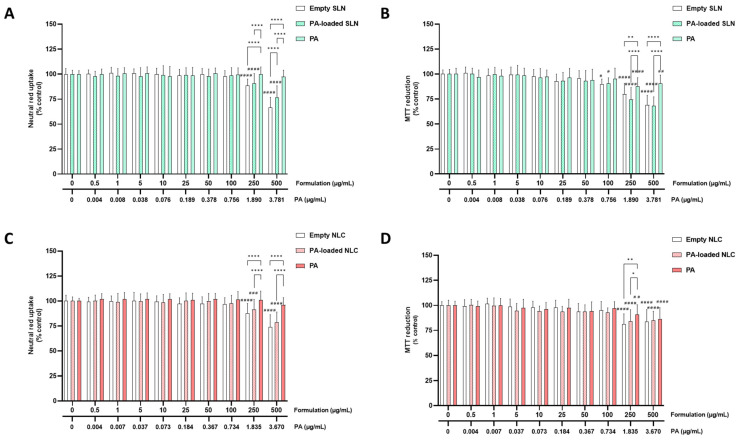
Cytotoxicity of the PA-loaded SLN and NLC, the corresponding concentrations of free PA, and empty SLN and NLC formulations, evaluated in differentiated SH-SY5Y cells by the neutral red uptake (**A**,**C**) and MTT reduction (**B**,**D**) assays, 24 h after exposure. Results are expressed as mean + SD from at least 4 independent experiences, performed in triplicate. Statistical comparisons were made using two-way ANOVA to compare means of different groups, followed by the Tukey’s multiple comparisons test (^#^ *p* < 0.05; ^##^ *p* < 0.01; ^###^ *p* < 0.001; and ^####^ *p* < 0.0001 for each formulation vs. 0 μg/mL; * *p* < 0.05; ** *p* < 0.01; and **** *p* < 0.0001 for comparisons between formulations, at each concentration). In all cases, *p* values < 0.05 were considered significant.

Figure 2 and Figure 3 show the results of the cytotoxicity evaluation of the AE- and PA-loaded SLN and NLC, free AE, free PA, and empty SLN and NLC formulations, in the differentiated SH-SY5Y cells, with the neutral red uptake (A,C) and MTT reduction (B,D) assays.

As shown in Figure 2A,C and Figure 3A,C, both the empty and AE- or PA-loaded SLN and NLC significantly decreased the viability of the differentiated SH-SY5Y cells when tested at concentrations equal or above 250 µg/mL, as evaluated by the significant decrease in the NR uptake, 24 h after exposure to 250 and 500 µg/mL of each formulation. For the free AE and PA, no significant cytotoxic effects were detected within the tested concentration range. Furthermore, when tested at the highest concentrations, significant differences were detected between the 250 and 500 µg/mL of the SLN and NLC formulations (both empty and AE or PA-loaded), when compared with the corresponding concentrations of the free AE and PA. The results of the biocompatibility studies of the SH-SY5Y cells showed that the developed empty and AE- and PA-loaded SLN and NLC formulations are safe, at least, for concentrations of up to 100 µg/mL and 24 h after exposure.

Regarding the MTT reduction assay, and as shown in Figure 2B,D and Figure 3B,D, similar results were obtained for the NLC formulations (empty and AE- and PA-loaded). In contrast, for the PA-loaded and empty SLN formulations, a small, but significant, decrease in the cell metabolic activity was observed (the MTT reduction significantly decreased to 89.7% and 90.8%, 24 h after exposure to the 100 µg/mL of empty SLN and PA-loaded SLN, respectively). Again, a significant decrease in the MTT reduction was also observed for all the the SLN formulations (empty, PA-loaded, and AE-loaded) when the formulations were tested at 250 and 500 µg/mL. Additionally, in some cases, significant differences were detected between the 250 and 500 µg/mL of the SLN and NLC formulations (both empty and AE and PA-loaded), and the corresponding concentrations of the free AE and PA. The slight difference found between both methods that were used to assess the cell viability (with the MTT reduction assay detecting significant differences for the 100 µg/mL PA-SLN and empty SLN formulations) can be explained by the fact that these methods use different principals to assess the cytotoxicity. According to the obtained results, the developed SLN and NLC formulations (empty and AE or PA-loaded) were safe towards the differentiated SH-SY5Y cells for concentrations equal to or below 100 µg/mL (% of cell viability is always above the 85% threshold). Accordingly, the 100 µg/mL was the concentration that was selected as the highest safe concentration of the SLN and NLC formulations (both empty and PA- or AE- loaded) that could be used in the subsequent studies on the differentiated SH-SY5Y cells, namely the studies evaluating the potential neuroprotective effects of the developed formulations and their capacity to be incorporated intracellularly.

#### 5.2.2. RPMI 2650 Cells

**Figure 4:**
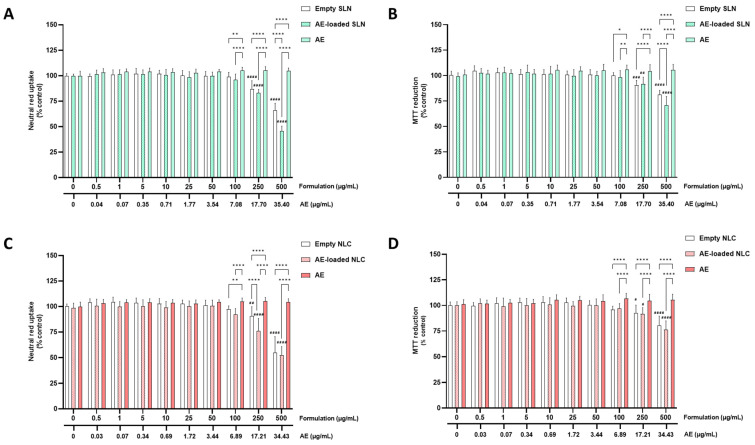
Cytotoxicity of the AE-loaded SLN and NLC, the corresponding concentrations of free AE, and empty SLN and NLC formulations, evaluated in RPMI 2650 cells by the neutral red uptake (**A**,**C**) and MTT reduction (**B**,**D**) assays, 24 h after exposure. Results are expressed as mean + SD from at least 4 independent experiences, performed in triplicate. Statistical comparisons were made using two-way ANOVA to compare means of different groups, followed by the Tukey’s multiple comparisons test (^#^ *p* < 0.05; *^##^ p* < 0.01; ^###^ *p* < 0.001 and ^####^ *p* < 0.0001 for each formulation vs. 0 μg/mL; * *p* < 0.05; ** *p* < 0.01; and **** *p* < 0.0001 for comparisons between formulations, at each concentration). In all cases, *p* values < 0.05 were considered significant.

**Figure 5:**
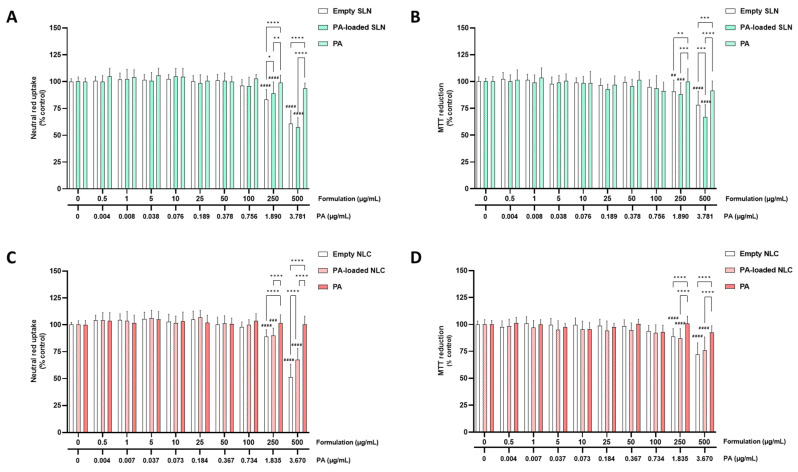
Cytotoxicity of the PA-loaded SLN and NLC, the corresponding concentrations of free PA, and empty SLN and NLC formulations, evaluated in RPMI 2650 cells by the neutral red uptake (**A**,**C**) and MTT reduction (**B**,**D**) assays, 24 h after exposure. Results are expressed as mean + SD from at least 4 independent experiences, performed in triplicate. Statistical comparisons were made using two-way ANOVA to compare means of different groups, followed by the Tukey’s multiple comparisons test (^##^ *p* < 0.01; ^###^ *p* < 0.001; and ^####^ *p* < 0.0001 for each formulation vs. 0 μg/mL; * *p* < 0.05; ** *p* < 0.01; *** *p* < 0.001; and **** *p* < 0.0001 for comparisons between formulations, at each concentration). In all cases, *p* values < 0.05 were considered significant.

Figure 4 and Figure 5 show the results of the cytotoxicity evaluations of the AE- and PA-loaded SLN and NLC, the free AE, free PA, and the empty SLN and NLC formulations, in the RPMI 2650 cells, with the neutral red uptake (A,C) and MTT reduction (B,D) assays.

In the RPMI 2650 cells, as shown in Figure 4A,C and Figure 5A,C, the cell viability was reduced when the cells were exposed to concentrations of 250 and 500 µg/mL of all the tested SLN formulations. The NR uptake significantly decreased to 83.1% and 61.0%, 24 h after exposure to 250 and 500 µg/mL of the empty SLN, respectively; to 88.9% and 57.4%, 24 h after exposure to 250 and 500 µg/mL of the PA-loaded SLN, respectively; and to 83.0% and 45.6%, 24 h after exposure to 250 and 500 µg/mL of the AE-loaded SLN, respectively. For the NLC formulations, cytotoxicity was also observed with the NR uptake, which significantly decreased to 89.1% and 51.2%, 24 h after exposure to 250 and 500 µg/mL of the empty NLC, respectively; to 88.9% and 67.3%, 24 h after exposure to 250 and 500 µg/mL of the PA-loaded NLC, respectively; and to 76.0% and 52.2%, 24 h after exposure to 250 and 500 µg/mL of the AE-loaded NLC, respectively. For the free PA and AE, no significant cytotoxic effects were detected within the tested concentration range. Therefore, significant differences were detected between the free PA or AE, and the SLN and NLC formulations (both empty and PA- or AE-loaded), at the highest tested concentrations.

In Figure 4B,D and Figure 5B,D the results of the evaluations of the formulations of the cytotoxicity towards the RPMI 2650 cells are presented, assessed by the MTT reduction assay. In agreement with the NR uptake assay, a significant decrease in the cell metabolic activity was observed for all the tested formulations at the concentrations of 250 and 500 µg/mL (empty and AE- or PA-loaded SLN and NLC formulations). Again, no significant cytotoxic effects were detected for the free PA and AE, within the tested concentration range.

The results of the cytotoxicity evaluation of the developed formulations (empty and AE- or PA-loaded SLN and NLC), and of the free PA and AE, for the RPMI 2650 cells, allowed for the conclusion that the encapsulation of AE and PA, either in NLC or in SLN, significantly decreased the cell viability at the highest tested concentrations (250 and 500 µg/mL), in agreement with the results that were obtained with the differentiated SH-SY5Y cells. Furthermore, and also in agreement with the results that were obtained for the differentiated SH-SY5Y cells, the developed formulations could be safely used in RPMI cells at concentrations of up to 100 µg/mL and for 24 h of exposure.

### 5.3. Evaluation of the Neuroprotective Effects

#### 5.3.1. Protective Effects against *t*-BHP-Induced Cytotoxicity

**Figure 6:**
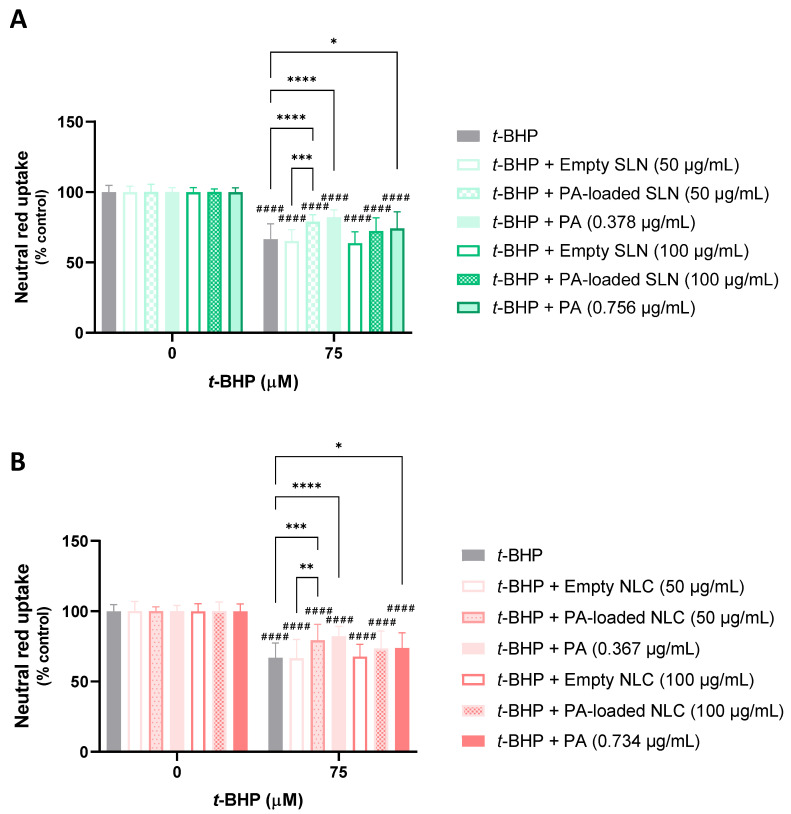
Potential neuroprotective effects of the developed SLN (**A**) and NLC (**B**) formulations (empty and PA-loaded), and free PA, against *t*-BHP-induced cytotoxicity, evaluated in differentiated SH-SY5Y cells, by the neutral red uptake assay, 24 h after exposure to the aggressor. Results are expressed as mean + SD from 4 independent experiences, performed in triplicate. Statistical comparisons were made using two-way ANOVA followed by the Šídák’s multiple comparisons test (^####^ *p* < 0.0001 versus control cells (0 µM t-BHP)) or by the Tukey’s multiple comparisons test (* *p* < 0.05; ** *p* < 0.01; *** *p* < 0.001; and **** *p* < 0.0001, for comparisons between formulations, PA and *t*-BHP alone, at each *t*-BHP concentration). In all cases, *p* values < 0.05 were considered significant.

Figure 6 shows the results of the potential neuroprotective effects of the PA-loaded and empty SLN and NLC, and the respective concentrations of the free PA, against *t*-BHP-induced cytotoxicity. According to the results, when tested alone, *t*-BHP (75 µM) significantly reduced the viability of the differentiated SH-SY5Y cells to ~67%, 24 h after exposure, and when compared to the control cells (0 µM). When the differentiated SH-SY5Y cells were exposed to 75 µM of *t*-BHP in the presence of the empty SLN formulations (50 and 100 µg/mL), the cells’ viability was 68.3% and 67.2%, respectively, evidencing no significant protective effects when compared to their exposure to the chemical aggressor alone (Figure 6A). Concerning the simultaneous exposure to 75 µM of *t*-BHP and 50 or 100 µg/mL of the PA-loaded SLN, the cell viability increased to 79.0% and 72.3%, respectively, demonstrating a significant protection of the differentiated SH-SY5Y cells against the *t*-BHP-induced cytotoxicity, only when the PA-loaded SLN formulation was tested at 50 µg/mL. When the free PA was tested, a significantly higher cell viability was observed, highlighting the potential of the compound to protect cells against *t*-BHP-induced oxidative stress. The NR uptake significantly increased to 82.1% and 74.1% when the differentiated SH-SY5Y cells were exposed to the chemical aggressor in the presence of 0.378 and 0.756 µg/mL of the free PA, respectively. When the cell viability was assessed after exposure to 75 µM of *t*-BHP in the presence of 50 or 100 µg/mL of the empty NLC, a cell viability of 66.6% and 67.7% was obtained, respectively, demonstrating no significant protective effects of the empty NLC against *t*-BHP-induced cytotoxicity (Figure 6B). On the other hand, when the exposure to the chemical aggressor occurred in the presence of 50 or 100 µg/mL of the PA-loaded NLC, the cell viability ascended to 79.3% and 73.4%, respectively. However, and in agreement with results that were obtained for the PA-loaded SLN formulation, a significant protective effect was only observed for the lowest concentration of the PA-loaded NLC (50 µg/mL). When the exposure to *t*-BHP occurred in the presence of 0.367 or 0.734 µg/mL of the free PA (the concentrations of the PA at 50 and 100 µg/mL of the PA-loaded NLC, respectively), and in agreement with the studies performed with the SLN formulations, the cell viability significantly increased to 82.2% and 73.8%, respectively, highlighting again the potential of astaxanthin in protecting neuronal cells against *t*-BHP-induced oxidative stress.

**Figure 7:**
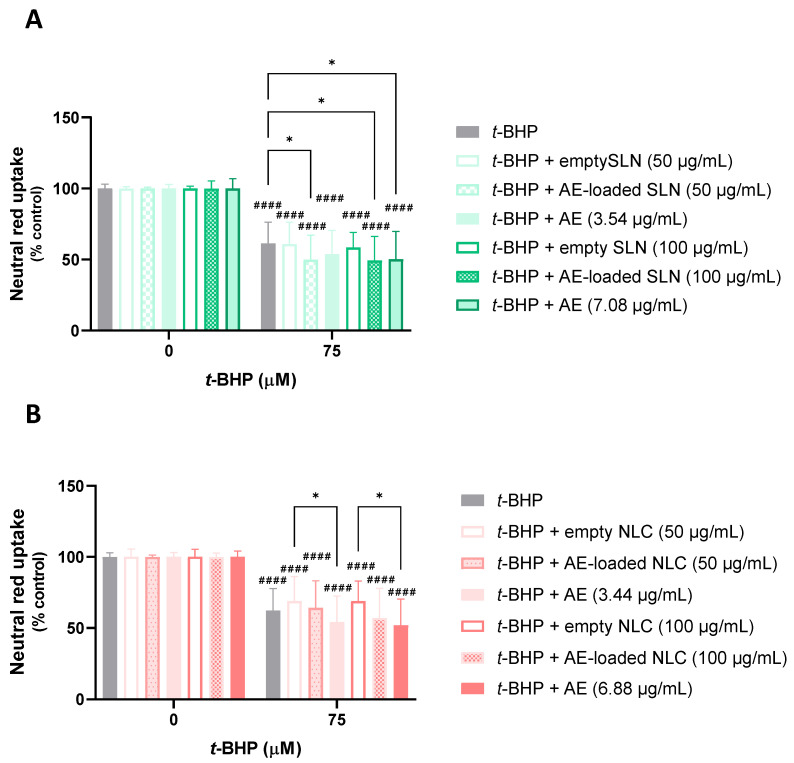
Potential neuroprotective effects of the developed SLN (**A**) and NLC (**B**) formulations (empty and AE-loaded), and free AE, against *t*-BHP-induced cytotoxicity, evaluated in differentiated SH-SY5Y cells, by the neutral red uptake assay, 24 h after exposure to the aggressor. Results are expressed as mean + SD from 4 independent experiences, performed in triplicate. Statistical comparisons were made using two-way ANOVA followed by the by the Šídák’s multiple comparisons test (^####^ *p* < 0.0001 versus control cells (0 µM t-BHP)) or by the Tukey’s multiple comparisons test (* *p* < 0.05, for comparisons between formulations, AE and *t*-BHP alone, at each *t*-BHP concentration). In all cases, *p* values < 0.05 were considered significant.

In Figure 7, the results that were obtained from the evaluation of the protective effects of the AE-loaded and empty NLC and SLN formulations, and the respective concentrations of the free AE against the *t*-BHP-induced cytotoxicity, are presented. It can be seen that the exposure of the differentiated SH-SY5Y cells to 75 µM of *t*-BHP significantly reduced the cell viability to ~62%, 24 h after exposure, and when compared to the control cells (0 µM). Similarly, the simultaneous exposure of the SH-SY5Y cells to the same concentration of *t*-BHP and to 50 µg/mL of the empty SLN or AE-loaded SLN formulations, or to 3.54 µg/mL of the free AE (the concentration of AE at 50 µg/mL of the AE-loaded SLN formulation), had no significant effect on the cell viability, when compared to the exposure to the chemical aggressor alone, with cell viability values of 60.9%, 49.8%, and 53.8%, respectively (Figure 7A). Similar results were observed after the exposure of the SH-SY5Y cells to 75 µM of *t*-BHP in the presence of 50 µg/mL of the empty NLC or AE-loaded NLC formulations, or at 3.44 µg/mL of the free AE, with a cell viability of 68.9%, 64.2%, and 54.2%, respectively (Figure 7B). Subsequent assays with a higher concentration of the formulations and free AE were performed. The results showed that simultaneous exposure to the same concentrations of *t*-BHP and to 100 µg/mL of the empty SLN or AE-loaded SLN formulations, or to 7.08 µg/mL of the free AE, also had no significant effect on the cell viability, when compared to the exposure to the chemical aggressor alone, with the percentage of the NR uptake being 58.4%, 49.3%, and 50.1%, respectively. The same was observed after the exposure of the SH-SY5Y cells to 75 µM of *t*-BHP in the presence of 100 µg/mL of the empty NLC or AE-loaded NLC, or 6.88 µg/mL of the free AE, with cell viability values of 69.0%, 57.1%, and 52.0%, respectively.

Overall, no significant protective effects against the *t*-BHP-induced cytotoxicity were observed for the AE, either in the free or encapsulated SLN and NLC formulations, contrary to the significant protective effects that were observed for the PA at the two concentrations that were tested of the free PA, and at the smaller concentration (50 µg/mL) of the PA-loaded SLN and NLC formulations.

The formulations with the PA demonstrated, through the *t*-BHP assay, a greater neuroprotective effect (Figure 6), which highlighs the potential of using SLN and NLC that are loaded with natural antioxidants for the management of neurodegenerative diseases.

PA-loaded SLN and NLC formulations, when tested at the smallest concentration of 50 µg/mL, significantly protected the differentiated SH-SY5Y cells against *t*-BHP-induced cell death. Furthermore, the free PA was capable of counteracting *t*-BHP-mediated oxidative stress, significantly increasing the cell viability at all the concentrations tested. Given the lack of significant protection afforded by the empty SLN and NLC formulations, the obtained results can be exclusively attributed to the astaxanthin’s antioxidant activity, which has the capability of neutralizing the deleterious effect of ROS.

#### 5.3.2. Protective Effects against MPP^+^-Induced Cytotoxicity

**Figure 8:**
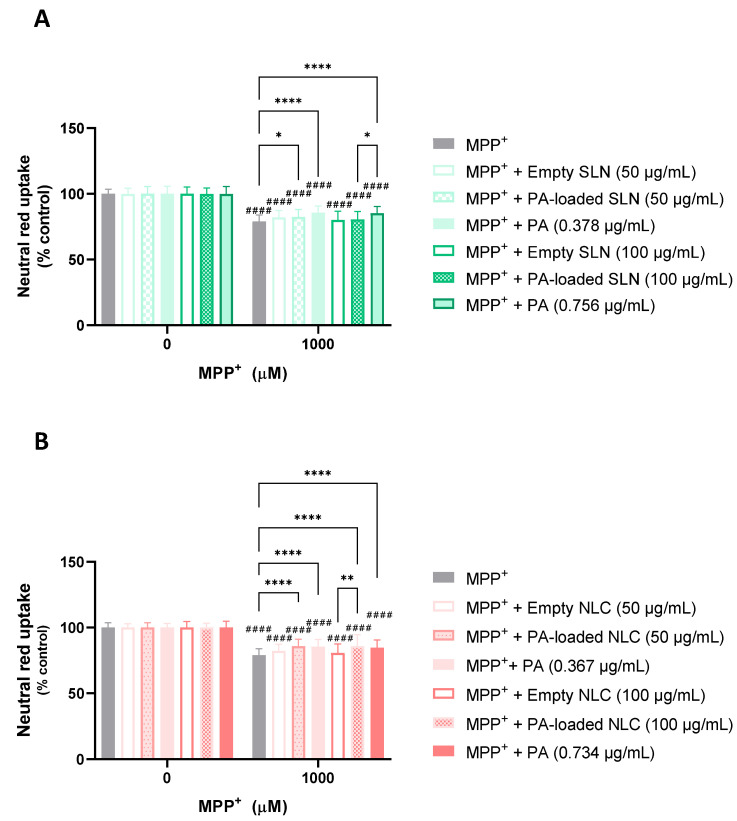
Potential neuroprotective effects of the developed SLN (**A**) and NLC (**B**) formulations (empty and PA-loaded), and free PA, against MPP^+^-induced cytotoxicity, evaluated in differentiated SH-SY5Y cells, by the neutral red uptake assay, 24 h after exposure to the aggressor. Results are expressed as mean + SD from 4 independent experiences, performed in triplicate. Statistical comparisons were made using two-way ANOVA followed by the Šídák’s multiple comparisons test (^####^ *p* < 0.0001 versus control cells (0 µM MPP^+^)) or by the Tukey’s multiple comparisons test (* *p* < 0.05; ** *p* < 0.01; and **** *p* < 0.0001, for comparisons between formulations, PA and MPP^+^ alone, at each MPP^+^ concentration). In all cases, *p* values < 0.05 were considered significant.

In Figure 8, the results that were obtained from the assessment of the potential neuroprotective effects of the PA-loaded and empty SLN and NLC are illustrated, as are the respective concentrations of the free PA, against MPP^+^-induced cytotoxicity. According to the obtained results, when tested alone, MPP^+^ (1000 µM) significantly reduced the viability of the differentiated SH-SY5Y cells to 78.9%, 24 h after exposure, and when compared to the control cells (0 µM). When the exposure to the chemical aggressor occurred in the presence of the 50 or 100 µg/mL of the empty SLN, no significant protective effects were observed (a cell viability of 82.1% and 80.1%, respectively). However, the PA-loaded SLN, only when tested at the lowest concentration (50 µg/mL), was capable of significantly counteracting MPP^+^-induced cell death, significantly increasing the cell viability to 82.5%. When the free PA was tested (0.378 and 0.756 µg/mL), significant protective effects were also observed for both the tested concentrations, with the NR uptake significantly increasing to 85.7% and 85.4%, respectively.

When the cell viability was assessed upon the exposure to MPP^+^ (1000 µM) in the presence of the 50 or 100 µg/mL of the empty NLC, no significant protective effects were observed (a cell viability of 82.0% and 80.7% was obtained, respectively). However, and outstandingly, when the exposure to the chemical aggressor occurred in the presence of the PA-loaded NLC (50 and 100 µg/mL), the cell viability significantly ascended to 85.8% and 85.9%, respectively, thus demonstrating the PA-loaded NLC-mediated protection against MPP^+^-induced cell damage. When the free PA was tested (0.367 and 0.734 µg/mL), and in agreement with the results that were obtained for the SLN formulations, the PA was capable of counteracting the MPP^+^-induced cytotoxicity, significantly increasing the cell viability to 85.5% and 84.6%, respectively.

**Figure 9:**
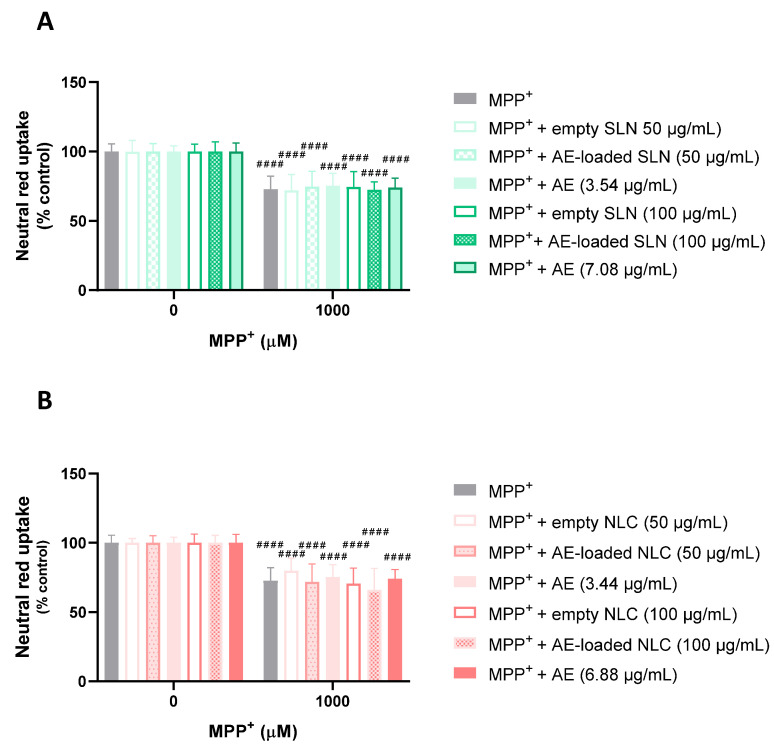
Potential neuroprotective effects of the developed SLN (**A**) and NLC (**B**) formulations (empty and AE-loaded), and free AE, against MPP^+^-induced cytotoxicity, evaluated in differentiated SH-SY5Y cells, by the neutral red uptake assay, 24 h after exposure to the aggressor. Results are expressed as mean + SD from 4 independent experiences, performed in triplicate. Statistical comparisons were made using two-way ANOVA followed by the Šídák’s multiple comparisons test (^####^ *p* < 0.0001 versus control cells (0 µM MPP^+^)) or by the Tukey’s multiple comparisons test (for comparisons between formulations, AE and MPP^+^ alone, at each MPP^+^ concentration). In all cases, *p* values < 0.05 were considered significant.

In Figure 9, the results that were obtained from the evaluation of the protective effects of the AE-loaded and empty NLC and SLN formulations are depicted, as are the respective concentrations of the free AE, against MPP^+^- induced cytotoxicity. It can be seen that the exposure of the differentiated SH-SY5Y cells to 1000 µM of MPP^+^ for 24 h significantly reduced the viability of the cells to 72.8%, when compared to the control cells (0 µM of MPP^+^). Similarly, the simultaneous exposure of the differentiated SH-SY5Y cells to the same concentration of MPP^+^ and to 50 µg/mL of the empty SLN or AE-loaded SLN formulations, or to 3.54 µg/mL of the free AE, had no significant effect on the cell viability, when compared to the exposure to the chemical aggressor alone, with NR uptake values of 72.0%, 74.7%, and 75.1%, respectively. Similar results were observed after the exposure of the SH-SY5Y cells to 1000 µM of MPP^+^ in the presence of a 50 µg/mL concentration of the empty NLC or AE-loaded NLC formulations, or to a 3.44 µg/mL concentration of the free AE, with the cell viability reaching the values of 79.8%, 71.8%, and 75.1%, respectively. Subsequent assays with higher concentrations of the formulations under study were performed. However, the simultaneous exposure of the differentiated SH-SY5Y cells to the same concentrations of MPP^+^ and to the 100 µg/mL concentration of the empty SLN or AE-loaded SLN formulations, or to a 7.08 µg/mL concentration of the free AE, also showed no significant protective effects on the cell viability, with NR uptake values of 74.6%, 72.3%, and 74.1%, respectively. The same was observed after the exposure of the differentiated SH-SY5Y cells to 1000 µM of MPP^+^ in the presence of 100 µg/mL of the empty NLC or AE-loaded NLC formulations, or to 6.88 µg/mL of the free AE, with cell viability values of 70.6%, 66.1%, and 74.1%, respectively.

The overall analysis of the results that were obtained in the present study (Figure 8) clearly demonstrated that the PA-loaded SLN, only when tested at the smallest concentration (50 µg/mL), was capable of slightly but significantly reducing the cytotoxicity that was induced by MPP^+^, 24 h after exposure. Notably, for the PA-loaded NLC, remarkable protective effects were observed for both the tested concentrations (50 and 100 µg/mL), clearly demonstrating the capacity of the PA-loaded NLC to counteract MPP^+^-mediated cell death. Similar results were obtained for the free PA, with significant protective effects being observed at all the tested concentrations. As no protective effects were observed upon the exposure to the empty SLN or NLC, the obtained positive outcomes for the PA- loaded NLC and SLN in counteracting the deleterious effects induced by this harmful neurotoxin can be attributed to the antioxidant potential of PA.

#### 5.3.3. Protective Effects against Iron (III)-Induced Cytotoxicity

**Figure 10:**
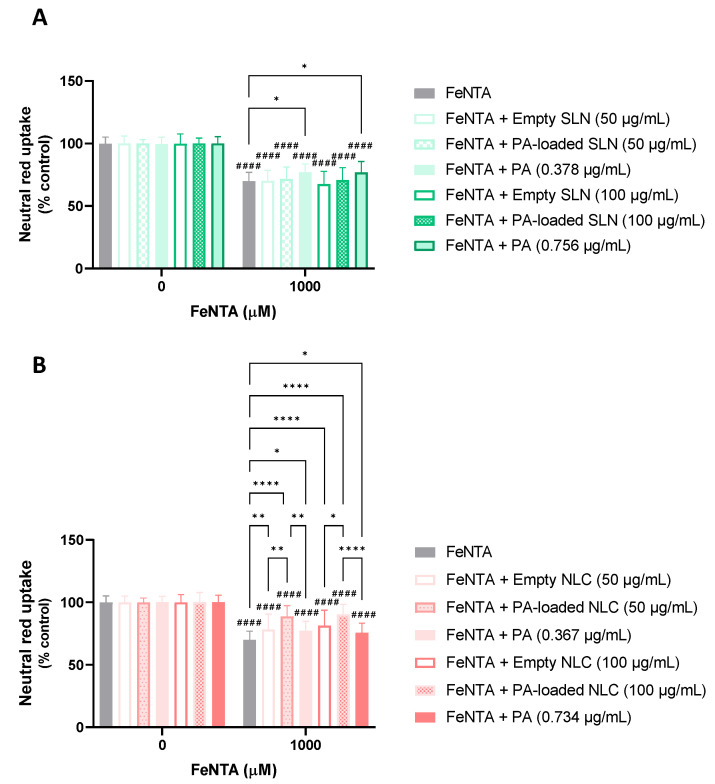
Potential neuroprotective effects of the developed SLN (**A**) and NLC (**B**) formulations (empty and PA-loaded), and free PA, against FeNTA-induced cytotoxicity, evaluated in differentiated SH-SY5Y cells, by the neutral red uptake assay, 24 h after exposure to the aggressor. Results are expressed as mean + SD from 4 independent experiences, performed in triplicate. Statistical comparisons were made using two-way ANOVA followed by the Šídák’s multiple comparisons test (^####^ *p* < 0.0001 versus control cells (0 µM FeNTA)) or by the Tukey’s multiple comparisons test (* *p* < 0.05; ** *p* < 0.01; and **** *p* < 0.0001, for comparison between formulations, PA and FeNTA alone, at each FeNTA concentration). In all cases, *p* values < 0.05 were considered significant.

In Figure 10, the results that were obtained in the assessment of the neuroprotective effects of the SLN and NLC formulations, both PA-loaded and empty, are shown, as are the respective concentrations of the PA alone, against iron(III)-induced cytotoxicity. The present study was not performed for the AE-loaded SLN and NLC formulations, and for the free AE, given the lack of significant protective effects that were observed in the previous studies.

According to the obtained results, when tested alone, the iron overload promoted by the FeNTA (1000 µM) significantly reduced the viability of the differentiated SH-SY5Y cells to ~70%, 24 h after exposure, and when compared to the control cells (0 µM). Furthermore, when the SLN formulations were tested, no significant protective effects against FeNTA-induced cytotoxicity were observed. Indeed, when exposed to 1000 µM FeNTA in the presence of the empty SLN formulations (50 and 100 µg/mL), the cells’ viability significantly decreased to 70.2% and 67.6%, respectively. Concerning the exposure to 1000 µM FeNTA in the presence of the PA-loaded SLN (50 and 100 µg/mL), the cell viability significantly decreased to 71.7% and 70.7%, respectively. However, significant protective effects against the FeNTA-induced cytotoxicity were observed when the exposure to the chemical aggressor occurred in the presence of the PA alone (0.378 and 0.756 µg/mL, corresponding to the concentrations of the PA in the 50 and 100 µg/mL of the PA-loaded SLN, respectively), with the cell viability significantly increasing up to ~77% for both the PA concentrations, when compared to the exposure to the chemical aggressor alone.

Outstandingly, when the FeNTA-induced cytotoxicity was assessed in the presence of the NLC formulations (50 and 100 µg/mL), remarkable protective effects were observed for all the tested conditions (empty NLC, PA-loaded NLC, and free PA). Indeed, when the exposure to the chemical aggressor occurred in the presence of the 50 and 100 µg/mL of the empty NLC, the cell viability significantly increased to 78.2% and 81.3%, respectively. In contrast, upon simultaneous exposure to FeNTA and the 50 or 100 µg/mL of the PA-loaded NLC, the cell viability significantly ascended to 88.6% and 90.2%, respectively. When the free PA was tested (0.367 and 0.734 µg/mL), the cell viability significantly increased to 77.3% and 75.6%, respectively. It is important to highlight that significant differences were observed between the empty and PA-loaded NLC formulations, with the latter being significantly more effective in protecting the differentiated SH-SY5Y cells against FeNTA-induced cytotoxicity. Furthermore, and outstandingly, the PA-loaded NLC was significantly more protective against the FeNTA-induced cytotoxicity, at both of the tested concentrations, when compared to the free PA. The protective effects afforded by the empty NLC may be justified by the antioxidant effect of vitamin E that is present in the composition of the NLC formulations, which was already reported to protect against iron-induced toxicity [71].

The overall analysis of the data that were obtained in the present study (Figure 10) demonstrated that the empty SLN (50 and 100 µg/mL) and PA-loaded SLN (50 and 100 µg/mL) formulations did not show significant protection against iron (III)-induced cytotoxicity. In contrast, the free PA (0.378 and 0.756 µg/mL) achieved a slight, but significant, protection against the aggressor. Regarding the NLC formulations, all the conditions that were tested (empty NLC 50 and 100 µg/mL); PA-loaded NLC (50 and 100 µg/mL); and free PA (0.367 and 0.734 µg/mL)) presented a significant protective effect towards iron (III)-induced cytotoxicity. In both cases, the neuroprotective effects of the free PA may have been due to its antioxidant properties.

### 5.4. Astaxanthin Cellular Uptake

**Figure 11:**
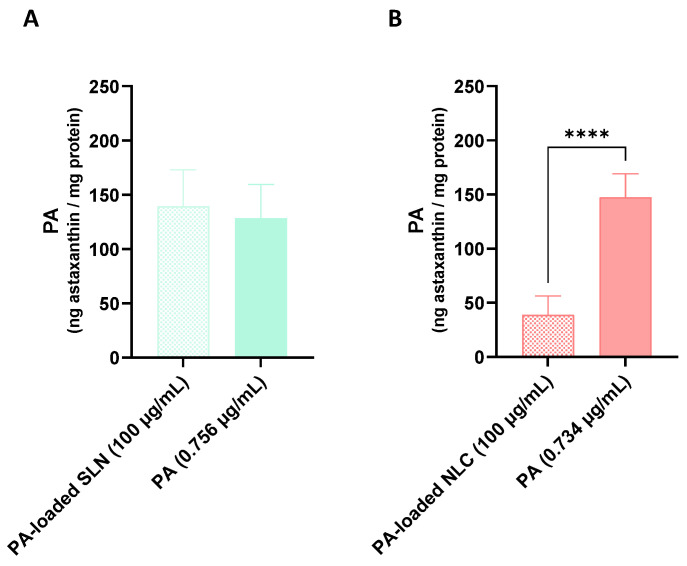
Cellular uptake of pure astaxanthin (PA), evaluated in differentiated SH-SY5Y, 24 h after exposure to 100 µg/mL of PA-loaded SLN (**A**) and NLC (**B**) formulations, as well as to the corresponding free PA concentrations. Results are presented as mean + SD from 4 independent experiments, performed in triplicate. Statistical comparisons were made using unpaired *t* test (**** *p* < 0.0001 for comparisons between free PA and PA-loaded formulations). In all cases, *p* values < 0.05 were considered significant.

As observed in Figure 11, the exposure of the differentiated SH-SY5Y cells to the PA-loaded SLN formulation (100 µg/mL) led to an intracellular accumulation of PA of 139.4 ng PA/mg protein, similar to the amount that was incorporated by the cells upon their exposure to the corresponding concentration of the free PA (0.756 µg/mL) (128.3 ng PA/mg protein). In contrast, the exposure of the differentiated SH-SY5Y cells to the PA-loaded NLC formulation (100 µg/mL) for 24 h resulted in the cellular uptake of 38.7 ng PA/mg protein, an amount that was significantly smaller than that observed upon exposure to the corresponding concentration of the free PA (0.734 µg/mL) (147.3 ng PA/mg protein).

Given the lack of significant neuroprotective effects of the AE-loaded SLN and NLC formulations, the cellular uptake of the astaxanthin that was present in the AE was not assessed.

## 6. Discussion

As presented in the results, AE and PA were successfully encapsulated in SLN and NLC, which showed a suitability for nose-to-brain administration, with respect to their parameters of particle size, PDI, and ZP. In more detail, all the particle size values were <200 nm with a PDI of <0.300, showing the adequacy for intranasal administration [68,69]. In relation to the ZP, all the formulations presented values close to |30| mV, suggesting a long-time stability [70]. The values that were obtained for the PDI and ZP are in agreement with those of other studies with astaxanthin-loaded lipid nanoparticles, which showed a homogeneous size distribution and stability during storage. For example, Tamjidi et al. prepared astaxanthin-loaded NLC formulations with PDI and ZP values in the range of 0.198–0.300 and −21.9 to −34.6 mV, respectively. Similar results were obtained by Li et al., who prepared astaxanthin-loaded SLN formulations with PDI and ZP values of 0.19 ± 0.042 and −36.2 ± 2.6 mV, respectively [66,72].

Stability studies showed greater stability during 180 days of storage (at 4 °C and 20 °C) for the empty SLN and NLC and the AE-loaded SLN and NLC, compared with the PA-loaded SLN and NLC, which showed stability up to 30 days of storage at 20 °C. Santonocito D. et al. obtained similar results with astaxanthin-loaded SLN, which showed an acceptable stability after 180 days of storage at room temperature [73]. Different results were obtained in another study, where astaxanthin-loaded SLN showed a better stability when stored at 4 °C, compared with the formulations that were stored at room temperature [72]. Rodriguez-Ruiz et al. also observed that astaxanthin-loaded NLC stored at 4 °C remained stable for 30 days [29], while Tamjidi et al. observed no significant changes in the particle size for the astaxanthin-loaded NLC that was stored at room temperature, also demonstrating that the particles remained stable when stored at 35 °C [74].

Regarding the EE results, they evidenced the high efficiency of SLN and NLC to encapsulate both types of astaxanthin (AE and PA). Other researchers have also obtained EE values of astaxanthin in lipid nanoparticles that were higher than 90%, which may be related to the lipophilic nature of the compound that provides affinity for the lipid matrix. In general, the incorporation of lipophilic molecules into lipid nanoparticles leads to high EE values [40,73,75]. The biocompatibility studies of the AE-, PA-loaded, and empty SLN and NLC formulations, and the free AE and PA, evaluated in nasal (RPMI 2650) and neuronal (SH-SY5Y) cells, showed the safety of the formulations for concentrations up to 100 µg/mL. Previous studies have reported similar results with these types of formulations, with the free drug being less toxic towards the differentiated SH-SY5Y cells, when compared with the formulations themselves. For example, Costa et al. observed a concentration-dependent cytotoxic effect with two different formulations (F1C1 and F2A8) of diazepam-loaded NLC over the concentration range tested (up to 500 μg/mL for F1C1 and up to 100 μg/mL for F2A8). In addition, the researchers observed that free diazepam was the least cytotoxic on the SH-SY5Y cells [40]. In contrast, Jojo et al. observed similar cytotoxicity with pioglitazone-loaded NLC and with free pioglitazone at a concentration of 10 μg/mL [76].

No cytotoxicity studies of the lipid nanoparticles that contained astaxanthin or the free compound in RPMI 2650 cells were found in the literature. However, some studies with lipid nanoparticle formulations containing other encapsulated drugs have shown a high cell viability towards RPMI 2650 cells. For example, in a study conducted by Cunha et al., in which in situ thermosensitive nasal gels were developed with NLCs containing rivastigmine for intranasal administration, in vitro studies were performed to evaluate the biocompatibility of the drug and the formulations containing the drug in the RPMI 2650 cells. The results showed that the rivastigmine solution and rivastigmine-loaded NLC formulation did not cause cytotoxicity in the concentration range of 25–1200 µg/mL and 13–203 µg/mL, respectively. Furthermore, the drug-free NLC formulation was demonstrated to be less cytotoxic to cells compared with the rivastigmine-loaded NLC formulation, as it was shown to be safe for the cells in the concentration range of 12–404 µg/mL [77].

Nonetheless, the outcomes that were observed in our study are not in agreement with some studies that have shown cell viability after astaxanthin exposure up to a concentration of 10 μg/mL. However, these studies used HepG2 and MCF-7 cells to test the formulations, which are tumour cells that are more resistant to the compound toxicity. Furthermore, in these studies, astaxanthin was not encapsulated, and the possible cytotoxicity of the other components of the formulation was not taken into account [78,79].

The results of the studies evaluating the neuroprotective effects of the developed formulations showed the superiority of the PA-loaded SLN and NLC in combatting oxidative stress, when compared with the AE-loaded SLN and NLC. In fact, the PA-loaded SLN and NLC formulations showed encouraging antioxidant effects, with a protective effect against ROS-induced neurotoxicity, and, for that reason, deserve to be further tested for their therapeutic potential in the management of neurodegenerative diseases. Furthermore, remarkably enhanced protective effects were observed when the PA was incorporated into NLC compared to the PA incorporated into SLN. This evidence should be highlighted and putatively attributed to the combined antioxidant effects of the PA and vitamin E. In reinforcement of this evidence, protective effects were observed with the empty NLC and attributed to the antioxidant effect of vitamin E that is present in the composition of the NLC formulations (please, see Table 1) [71]. The reasons for the AE’s lack of protection may be related to its composition (please, see Section 2), which includes oleic sunflower oil. Under the experimental conditions that were used, this oil can easily oxidize, leading to the formation of highly reactive species such as alkyl and peroxyl radicals, which can promote the degradation of astaxanthin [80].

The evaluation of the potential neuroprotective effects of astaxanthin has already been reported. For instance, Lee et al. evaluated the potential of using astaxanthin against the mitochondrial dysfunction induced by MPTP/MPP^+^. The experiments were performed in vivo, with C57BL/6 mice, and in vitro, with SH-SY5Y human neuroblastoma cells. The results of the in vitro studies showed that astaxanthin inhibited the apoptotic process that was induced by MPP^+^ in the SH-SY5Y cells, evidencing the antioxidant activity of the compound. Furthermore, it was observed from the in vivo studies that the neuronal loss that was induced by the MPTP exposure was significantly reduced after the astaxanthin administration. From these results, the researchers suggested that this compound was capable of preventing the cell death caused by ROS overproduction [81]. In another study, Liu et al. tested the use of astaxanthin as an inhibitor of ROS toxicity in SH-SY5Y cells. The ROS inducers that were used were docosahexaenoic acid hydroperoxide and 6-hydroxydopamine, which resulted in significant cell death. Outstandingly, the pre-treatment with astaxanthin prevented the aggressor-mediated cell death and significantly inhibited the apoptosis, ROS generation, and mitochondrial abnormalities caused by the two neurotoxins. These results suggested that the neuroprotective effects of astaxanthin rely on its mitochondria protection and antioxidant potential [82].

The results of the PA cellular uptake suggest that, contrary to the PA-loaded SLN, the PA-loaded NLC tended to stay more on the cell surface rather than penetrating. To support this conclusion, a greater neuroprotective effect against the harmful effects of all the tested chemical aggressors was observed with the PA-loaded NLC, which may be related to the formation of a protective barrier on the cell surface that prevented the entry of the aggressor agents. In more detail, although the uptake of the free PA and PA-loaded SLN was not significantly different, a decreased intracellular uptake of the compound was observed upon its encapsulation in NLC (PA-loaded NLC). However, despite this decrease in the PA cellular uptake, significantly higher protective effects were observed for the PA-loaded NLC when compared to the free PA. For example, this was seen for iron-induced cytotoxicity (see Section 5.3.3), which may be attributed to the combined protective effect of the PA and vitamin E that are both present in the formulation. Furthermore, since the free PA is very susceptible to degradation, its encapsulation in lipid nanoparticles has been proposed to improve their stability and promote targeting [26].

## 7. Conclusions

In this work, it was possible to develop four different formulations of lipid nanoparticles (SLN or NLC) containing astaxanthin from different sources (AE from the algae *Haematococcus pluvialis* and PA from the fungi *Blakeslea trispora*), with adequate particle size, PDI, ZP, and EE for nose-to-brain administration.

In vitro studies of differentiated SH-SY5Y cells and RPMI 2650 cells demonstrated that the developed formulations were safe for concentrations of up to 100 µg/mL and 24 h after exposure. Regarding the neuroprotection studies, the results that were obtained showed that the developed PA-loaded SLN and NLC formulations were capable of counteracting some of the pathophysiological mechanisms underlying neurodegeneration, such as oxidative stress. However, when compared to the PA-loaded SLN, the PA-loaded NLC formulation showed more efficient neuroprotective effects against the cytotoxicity that was induced by the tested aggressors. Additionally, for AE, either free or encapsulated in SLN or NLC, no significant neuroprotective effects were observed.

Nevertheless, it is mandatory to perform more studies to confirm the potential of this neuroprotection, such as an evaluation of the protective effects against lipid peroxidation, a quantification of the intracellular ROS levels, or an evaluation of the activity of antioxidant enzymes, such as SOD, acetylcholinesterase, and catalase, to further elucidate the mechanism(s) underlying the observed neuroprotection. Moreover, and considering the intranasal route that is proposed for the developed formulations, permeability studies of RPMI 2650 cells should be performed to confirm the suitability of the formulations for nose-to-brain administration.

In conclusion, keeping in mind that oxidative stress is related to several neurodegenerative diseases, and considering the antioxidant potential that was observed for the PA-loaded NLC formulation, its use may improve the treatment of these diseases, although in vivo experiments with animal models and clinical studies should be performed to further confirm this application.

## Figures and Tables

**Table 1:** Composition (%, *w*/*w*) of the developed SLN and NLC formulations, empty and loaded with astaxanthin extract (AE) and pure astaxanthin (PA).

Composition	(%, *w*/*w*)					
	Empty NLC	AE_NLC	PA_NLC	Empty SLN	AE_SLN	PA_SLN
Precirol^®^ 5 ATO	6.700	6.300	6.300	9.600	8.600	8.600
Vitamin E	2.900	2.700	2.700	-	-	-
AE	-	1.000	-	-	1.000	-
PA	-	-	0.1	-	-	0.1
Tween^®^ 80	4.200	4.200	4.200	4.200	4.200	4.200
Sodium deoxycholate	0.300	0.300	0.300	0.300	0.300	0.300
Benzalkonium chloride	0.020	0.020	0.020	0.020	0.020	0.020
Glycerin	0.003	0.003	0.003	0.003	0.003	0.003
Ultrapure water	q.s. 100	q.s. 100	q.s. 100	q.s. 100	q.s. 100	q.s. 100

**Table 2:** Particle size (Z-Ave), polydispersity index (PDI), and zeta potential (ZP) of the SLN and NLC formulations, empty and loaded with astaxanthin extract (AE) and pure astaxanthin (PA). Measurements were performed on days 0, 30, and after 180 days of storage at 4.0 ± 0.5 °C and 20.0 ± 0.5 °C.

Formulation	Day	Temperature (°C)	Z-Ave (nm)	PDI	ZP (mV)
Empty SLN	0	-	98.043 ± 1.200	0.212 ± 0.006	−20.033 ± 0.643
	30	4.0 ± 0.5	86.977 ± 0.233	0.237 ± 0.010	−23.867 ± 0.987
		20.0 ± 0.5	85.893 ± 0.571	0.217 ± 0.002	−20.867 ± 0.462
	180	4.0 ± 0.5	98.997 ± 0.945	0.246 ± 0.002	−20.667 ± 0.643
		20.0 ± 0.5	98.107 ± 0.641	0.230 ± 0.008	−22.700 ± 0.361
AE_SLN	0	-	106.967 ± 2.515	0.220 ± 0.017	−24.133 ± 0.379
	30	4.0 ± 0.5	104.133 ± 0.814	0.216 ± 0.008	−25.367 ± 1.677
		20.0 ± 0.5	101.133 ± 0.709	0.211 ± 0.007	−22.700 ± 1.058
	180	4.0 ± 0.5	105.167± 0.321	0.216± 0.009	−22.667 ± 0.416
		20.0 ± 0.5	104.367 ± 0.404	0.220 ± 0.008	−23.600 ± 0.520
PA_SLN	0	-	110.200 ± 6.482	0.349 ± 0.056	−24.333 ± 1.234
	30	4.0 ± 0.5	-	-	-
		20.0 ± 0.5	106.547 ± 9.360	0.457 ± 0.067	−29.800 ± 1.058
	180	4.0 ± 0.5	-	-	-
		20.0 ± 0.5	-	-	-
Empty NLC	0	-	109.033 ± 0.404	0.228 ± 0.006	−23.467 ± 1.079
	30	4.0 ± 0.5	113.767 ± 1.069	0.233 ± 0.008	−17.700 ± 1.652
		20.0 ± 0.5	116.700 ± 1.015	0.245 ± 0.011	−22.300 ± 0.265
	180	4.0 ± 0.5	119.567 ± 0.289	0.232 ± 0.002	−24.633 ± 0.416
		20.0 ± 0.5	116.033 ± 1.795	0.215 ± 0.004	−23.833 ± 0.462
AE_NLC	0	-	117.300 ± 2.163	0.222 ± 0.016	23.267 ± 0.451
	30	4.0 ± 0.5	119.533 ± 2.101	0.225 ± 0.003	25.367 ± 0.850
		20.0 ± 0.5	120.533 ± 0.723	0.234 ± 0.012	23.433 ± 0.252
	180	4.0 ± 0.5	126.833 ± 2.954	0.249 ± 0.010	−27.967 ± 0.643
		20.0 ± 0.5	174.167 ± 1.210	0.309 ± 0.009	23.333 ± 0.379
PA_NLC	0	-	97.610 ± 0.394	0.294 ± 0.035	−23.300 ± 1.058
	30	4.0 ± 0.5	-	-	-
		20.0 ± 0.5	109.000 ± 1.000	0.330 ± 0.021	−28.400 ± 0.954
	180	4.0 ± 0.5	-	-	-
		20.0 ± 0.5	-	-	-

## Data Availability

Not applicable.
